# Comparison of the pathway structures influencing the temporal response of salicylate and jasmonate defence hormones in *Arabidopsis thaliana*

**DOI:** 10.3389/fpls.2022.952301

**Published:** 2022-09-09

**Authors:** Erin A. Stroud, Jay Jayaraman, Matthew D. Templeton, Erik H. A. Rikkerink

**Affiliations:** ^1^The New Zealand Institute for Plant and Food Research Limited, Auckland, New Zealand; ^2^School of Biological Sciences, University of Auckland, Auckland, New Zealand; ^3^Bioprotection Aotearoa, Lincoln, New Zealand

**Keywords:** salicylic acid, jasmonic acid, phytohormone, defence, Arabidopsis, temporal regulation

## Abstract

Defence phytohormone pathways evolved to recognize and counter multiple stressors within the environment. Salicylic acid responsive pathways regulate the defence response to biotrophic pathogens whilst responses to necrotrophic pathogens, herbivory, and wounding are regulated *via* jasmonic acid pathways. Despite their contrasting roles *in planta*, the salicylic acid and jasmonic acid defence networks share a common architecture, progressing from stages of biosynthesis, to modification, regulation, and response. The unique structure, components, and regulation of each stage of the defence networks likely contributes, in part, to the speed, establishment, and longevity of the salicylic acid and jasmonic acid signaling pathways in response to hormone treatment and various biotic stressors. Recent advancements in the understanding of the *Arabidopsis thaliana* salicylic acid and jasmonic acid signaling pathways are reviewed here, with a focus on how the structure of the pathways may be influencing the temporal regulation of the defence responses, and how biotic stressors and the many roles of salicylic acid and jasmonic acid *in planta* may have shaped the evolution of the signaling networks.

## Introduction

Plants must defend themselves against multiple stressors in the environment. The induction and co-ordination of plant defence requires dynamic regulation. In the model plant *Arabidopsis thaliana* (hereafter referred to as Arabidopsis), hormones control various aspects of plant growth, development, and responses to internal and external stimuli. Several hormones, namely salicylic acid (SA), jasmonic acid (JA), ethylene (ET), and abscisic acid (ABA), regulate defence against biotic stressors, amongst other roles *in planta*. Defence against (hemi)-biotrophic pathogens, sucking insects, nematodes, and some viruses is regulated primarily *via* SA-dependent responses whilst necrotrophs and chewing insects are deterred by JA- and ET-dependent defence. Moreover, the JA defence network is segregated into two ‘branches’ of co-regulated genes controlled by the activity of specific transcription factors. The JA and ET co-regulated branch is controlled by the activity of ETHYLENE RESPONSE FACTOR (ERF) transcription factors to direct defence against necrotrophic pathogens, whereas the JA and ABA co-regulated branch is controlled by the activity of MYC transcription factors to regulate the response to large-scale tissue damage caused by insect and mammalian herbivory (Lorenzo et al., 2003, 2004).

**Figure 1:**
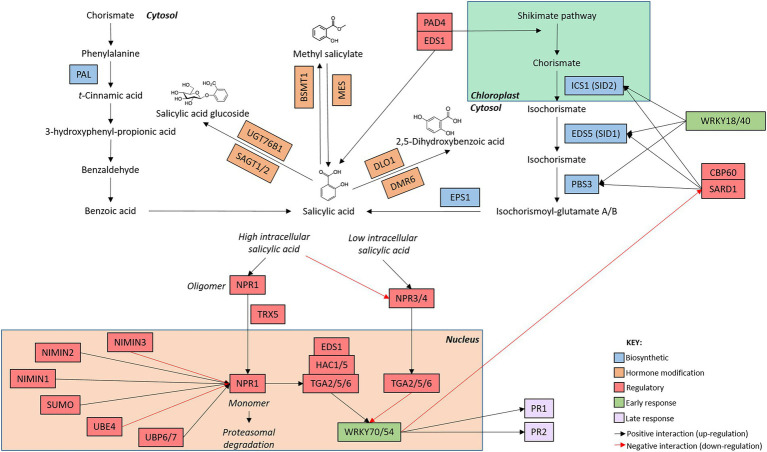
*Arabidopsis thaliana s*alicylic acid network map. The salicylic acid defence network is initiated with the induction of genes involved in hormone biosynthesis (blue boxes). Once salicylic acid has been produced, genes involved in the modification of salicylic acid (orange boxes) alter hormone structure. The production of metabolically active salicylic acid induces the expression of early (green boxes) and late (purple boxes) responsive genes. Regulatory genes (red boxes) modify the activity of genes involved at each stage of the signaling cascade. The intracellular location of the components of the signaling pathway are indicated by the bold text. Connections between genes are indicated by arrows whereby black arrow indicate positive interactions (upregulation) and red arrows indicate negative interactions (downregulation). While additional members of the pathway are known, only genes mentioned in this review are included in the network map.

**Figure 2:**
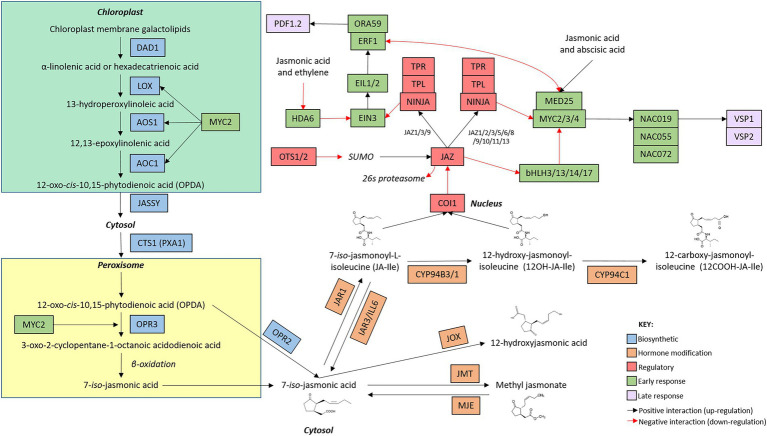
*Arabidopsis thaliana* jasmonic acid network map. The jasmonic acid defence network is initiated with the induction of genes involved in hormone biosynthesis (blue boxes). Once jasmonic acid has been produced, genes involved in the modification of jasmonic acid (orange boxes) alter hormone structure. The production of metabolically active jasmonic acid induces the expression of early (green boxes) and late (purple boxes) responsive genes. Regulatory genes (red boxes) modify the activity of genes involved at each stage of the signaling cascade. The intracellular location of the components of the signaling pathway are indicated by bold text. Connections between genes are indicated by arrows whereby black arrow indicate positive interactions (upregulation) and red arrows indicate negative interactions (downregulation). While additional members of the pathway are known, only genes mentioned in this review are included in the network map.

Hormones orchestrate the induction of complex networks of functionally linked genes, as demonstrated in the SA and JA network maps (Figures 1, 2). Signaling between the hormone networks, termed inter-pathway communication or crosstalk, provides an additional layer of defence regulation. Through positive and negative interactions between the defence networks, crosstalk controls the selective induction of the defence response best suited to countering the perceived stressor. Simultaneously, signaling pathways promoting an opposing response are inhibited (extensively reviewed by Kunkel and Brooks, 2002; Koornneef and Pieterse, 2008; Li et al., 2019; Aerts et al., 2021). Owing to their functional differences, the SA and JA defence networks are typically mutually antagonistic wherein the activation of one pathway will inhibit signaling *via* the other. However, large-scale additive and synergistic pathway communication has also been described (Mine et al., 2018; Hickman et al., 2019; Zhang et al., 2020). Furthermore, the two branches of the JA defence network are also antagonistic (Lorenzo et al., 2003, 2004). Specific genes within each defence response, termed crosstalk nodes, direct the activation of one pathway while actively disabling or dampening other pathways (Hickman et al., 2019). Collectively, crosstalk directs energy and resources towards the most appropriate defence response during stress events.

Despite its advantages, crosstalk may be deleterious in multi-attacker environments. In their natural setting, plants are challenged by diverse, and often simultaneous stressors. Crosstalk ensures that only one defence response pathway can be prioritized at a time, arming the plant to defend itself against the challenge at hand. However, due to the mutual antagonism observed between the defence networks, this enhanced defence against one stressor may be accompanied by the repression of antagonistic defence networks, leading to increased susceptibility to secondary stressors (Spoel et al., 2007; Vos et al., 2015). In such situations, the order and characteristics of each sequential stressor may affect the plant’s ability to defend itself against future attack (reviewed by Lazebnik et al., 2014). Additionally, several other hormones have been found to interact with the defence hormone pathways, including auxin, cytokinin, gibberellin and brassinosteroids. While multiple hormone inputs allow for the deployment of a highly regulated, attacker-specific defence response, trade-offs in plant defence and growth and development have been observed (Bostock, 2005; Robert-Seilaniantz et al., 2011; Huot et al., 2014).

Spatial regulation of defence signaling provides an additional layer of regulation allowing plants to respond to multiple, concurrent stressors. Upon simultaneous infection with a biotroph and a necrotroph, the subsequent trade-off in susceptibility and resistance was limited to the tissue immediately adjacent to the initial infection site (Spoel et al., 2007). This observation may be explained by recent research highlighting the ability of plants to spatially separate their defence responses under multi-stressor events. Here, infection of Arabidopsis with the biotrophic pathogen *Pseudomonas syringae* caused an upregulation of SA in infected cells, while ‘bystander’ cells surrounding the infection site upregulated the JA pathway, likely limiting the hypersensitive response to the infection zone only (Betsuyaku et al., 2018; Wolinska and Berens, 2019; Salguero-Linares et al., 2022).

Together with crosstalk and pathway segregation, these tissue-specific defence responses act collaboratively to create a dynamic and plastic network of defence, capable of responding to a changing environment.

## The phytohormone networks display unique expression profiles in response to exogenous treatment

Recent research has focused on characterizing the structure, genetic components, and temporal regulation of the phytohormone networks and defence induction. Time-series dense transcriptomic analysis of the response to exogenous phytohormone treatment has facilitated in-depth profiling and comparison of the SA and JA defence networks in Arabidopsis. In the sections below, focusing on Arabidopsis, we start by reviewing the networks induced by phytohormones and follow this with discussion in Section “Biotic stressors induce contrasting phytohormones and expression profiles”, where we compare this with induction by biotic agents that predominantly focus on either SA or JA.

### Phytohormone-induced SA defence network

**Figure 3:**
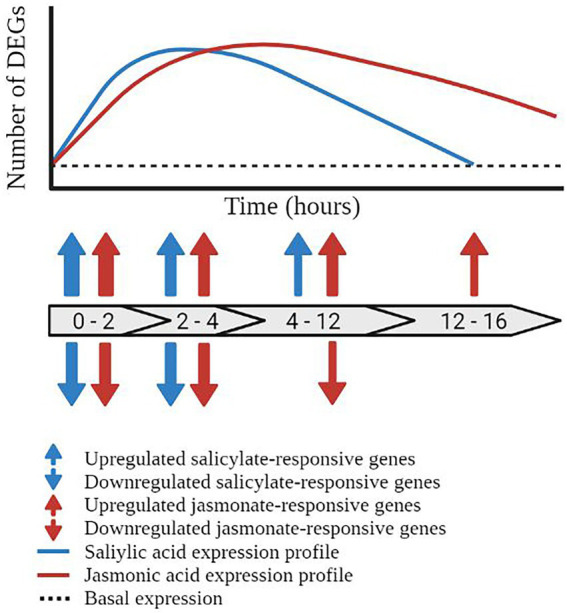
Temporal dynamics of the salicylic acid and jasmonic acid defence networks. Schematic representation of the temporal regulation of transcriptionally responsive genes in the salicylic acid (blue line) and jasmonic acid (red line) defence networks. Block arrows represent the number of hours post treatment (hpt). Prior to pathway induction, the plant displays a basal level of gene expression (black dashed line). Activation of the salicylic acid pathway results in the induction of defence-associated genes initiating at 0–2 hpt and returning to basal expression by 12–16 hpt. Blue arrows represent the upregulation and downregulation of salicylic acid-responsive differentially expressed genes (DEGs). Transcriptional reprogramming of the jasmonic acid defence network initiated within 0–2 hpt but does not return to basal expression levels within the 16 h time course, represented by red arrows. The thickness of the arrows represents the number of DEGs at the given time point.

The SA defence network undergoes rapid, dynamic, and transient transcriptional reprogramming following exogenous phytohormone or elicitor treatment. Recently, a meta-analysis of publically available expression profiles following treatment with SA or its chemical mimic benzothiadiazole (BTH) identified that the SA defence network was rapidly induced and transcriptionally dynamic over the 16 h timecourse assayed (Zhang et al., 2020). Similar to the findings described by Hickman et al. (2019) (which were not included in the meta-analysis), the transcriptomic response to SA treatment was rapid with the most significant changes in transcription (both upregulation and downregulation) observed between 0 and 2 h post-treatment (hpt). Additional ‘waves’ of transcriptional reprogramming were observed between 2–4 hpt and 4–12 hpt with a return to pre-induction, or basal expression levels (for most genes) by 12–16 hpt (Figure 3). Collectively, these studies indicate that the phytohormone-induced SA defence network operates in a rapid, dynamic, and temporally limited signaling network with the majority of signaling occurring in distinct ‘waves’ of transcriptional reprogramming between the 0.5 and 12 hpt timecourse (Hickman et al., 2019; Zhang et al., 2020).

### Phytohormone-induced JA defence network

The JA defence network displayed a rapidly induced and (compared to the SA responses) a more prolonged expression profile in response to exogenous phytohormone or elicitor treatment. A similar meta-analysis assaying the response to methyl-jasmonate (MeJA, a derivative of JA) or coronatine (a mimic of JA), over a 16 h timecourse was generated by Zhang et al. (2020), allowing direct comparison of the SA and JA phytohormone networks. The response to methyl-jasmonate and coronatine was rapid with upregulation of the JA pathway observed within 1 hpt. Similar results were observed by Hickman et al. (2017) wherein the majority of MeJA-responsive genes were transcriptionally active by 2 hpt. While some fluctuations in the number of transcriptionally responsive genes were observed across the timecourse (namely at 2, 6, and 10 hpt in the MeJA treated samples), these genes demonstrated a prolonged pattern of upregulation, extending to 16 hpt in the response to MeJA and 24 hpt in the response to coronatine (Zhang et al., 2020; Figure 3). Collectively, these studies highlight the prolonged and rapidly induced expression profile of the JA defence network in response to MeJA and coronatine. An extension of the assayed time points past 16 hpt would give greater insight into the genetic reprogramming during defence, and presumably would see a return to basal expression in the JA network.

To date, most studies have used exogenous hormone treatments or toxins such as coronatine to characterize the phytohormone networks. It is possible that these treatments are perceived differently and the hormone mimics not able to be metabolized or inactivated by the plant in the same manner as endogenously produced hormones, therefore potentially inducing different transcriptional responses *in planta*. Some caution is necessary when applying the findings of these studies to those focusing on the response to biotic and abiotic stressors, which are likely to vary in the temporal profile and genetic components of defence induction and signaling.

## Biotic stressors induce contrasting phytohormones and expression profiles

By comparing the transcriptomic response to pests and diseases with different lifestyles, common trends can be observed between the expression profiles elicited by exogenous hormone treatment and biotic attack (Supplementary Table 1). This review focuses on effective plant response to pathogen and insect attack, therefore the scope of the following papers is limited to those investigating resistant host-microbe and host-insect interactions.

### Response to biotrophic pathogens

Biotrophic pathogens colonize their hosts to derive nutrients from living cells, typically causing less damage to the plant than necrotrophic pathogens that promote extensive cell death (see below). The SA defence response is typically induced following infection with avirulent biotrophic pathogens. The bacterial pathogen *Pseudomonas syringae* pv. *tomato* DC3000 (*Pto*) and the oomycete *Hyaloperonospora arabidopsidis* (*Hpa*) are commonly used to investigate host-biotroph interactions in Arabidopsis. Infection with avirulent (i.e., non-disease inducing) strains of *Pto* AvrRpt2 and/or AvrRpm1 induced largescale transcriptional reprogramming over a 24 h timecourse; initiating at 3 h post infection (hpi) before peaking between 4 and 9 hpi and returning to near-basal levels of expression by 24 hpi (Mine et al., 2018). A similar expression profile was observed by De Vos et al. (2005) wherein infection with avirulent *Pto* AvrRpt2 induced the expression of the antimicrobial defence gene *PATHOGENESIS-RELATED PROTEIN 1* (*PR1*) commonly used as a marker of the SA defence network at 12 hpi. *PR1* then gradually declined in expression over the following 48–72 h period. Hormone accumulation in response to infection with avirulent *Pto* AvrRpt2 followed a similar pattern to the transcriptomic response. SA accumulation was detected within 3 hpi before peaking at 24 hpi and returning to basal levels at 48 hpi in response to *Pto* (De Vos et al., 2005). Similarly, infection with *Hpa* Emoy2 in resistant Arabidopsis Col-0 plants induced a similar transcriptomic response as *Pto,* with the upregulation of PR1 observed by 24 hpi. Expression of PR1 was detected at 72 hpi and returned to basal levels by 120 hpi (Asai et al., 2014).

The response to biotrophic pathogens and exogenous SA treatment appears to follow a similar expression profile (Figure 3). Defence induction was observed within 3 hpi for *Pto* and within 24 hpi for *Hpa.* Despite its rapid induction, this upregulation was not sustained with reduced expression observed by 24–72 hpi for *Pto* and ~72 hpi for *Hpa*. Although the response to biotrophic pathogens occurred over a delayed timecourse, presumably due to the requirement of the plant to detect infection, the response to biotic stressors displayed a similar expression profile as the response to exogenous SA application with a rapid induction followed by the deactivation of signaling within a relatively short timeframe.

### Response to necrotrophic pathogens

Necrotrophic pathogens invade and kill host cells to gain nutrients, typically causing significant tissue damage to the host. Necrotrophic pathogens typically induce the defence network co-regulated by JA and ET. The model necrotrophic fungal pathogens *Alternaria brassicicola* and *Botrytis cinerea* are often used to investigate host-necrotroph interactions in Arabidopsis. In response to *A. brassicicola* infection, upregulation of the JA- and ET-induced anti-fungal defence marker gene *PLANT DEFENSIN 1.2* was observed as early as 12 hpi (van Wees et al., 2003) and remained upregulated at 36 hpi (van Wees et al., 2003), 48 hpi (Penninckx et al., 1996; De Vos et al., 2005), 72 hpi (Mukherjee et al., 2009), and 96 hpi (Penninckx et al., 1996), albeit across different studies. Hormone and protein accumulation followed a similar trend as the transcriptomic response. The accumulation of JA following infection initiated at 12 hpi and continued to increase at the final time point assayed, 72 hpi (De Vos et al., 2005). The accumulation of *PLANT DEFENSIN* protein was first detected at 50 hpi before peaking at ~75 hpi and remaining stable at 100 hpi (Penninckx et al., 1996). The response to *B. cinerea* followed a similar expression profile. Upregulation of defence marker genes and transcriptomic reprogramming occurred as early as 14–18 hpi, with some variability between studies. The expression of the marker genes typically increased by ~24 hpi and remained stable at 40–48 hpi, the final time points assayed (Ferrari et al., 2003; Birkenbihl et al., 2012; Windram et al., 2012; Coolen et al., 2016; Veillet et al., 2017).

The transcriptomic response to necrotrophic pathogens appears to follow a similar expression profile as the response to exogenous JA treatment (Figure 3). The upregulation of defence marker genes was observed as early as 12–14 hpi in the response to *A. brassicicola* and *B. cinerea* (van Wees et al., 2003; Birkenbihl et al., 2012; Windram et al., 2012). Once induced, defence signaling and marker gene upregulation remained upregulated for the entirety of the experimental time points assay, stretching to 48 hpi for *B. cinerea* assays (Ferrari et al., 2003; Birkenbihl et al., 2012; Windram et al., 2012), and 96 hpi for *A. brassicicola* assays (Penninckx et al., 1996). This observation is similar to the expression profile observed in response to MeJA or coronatine application, wherein the transcriptomic response is rapidly induced and remains stably upregulated over the assayed timecourse. The lack of continuity between studies, in regards to both time points used and infection methodology, may impact the collective findings of the studies and therefore should be considered in future work.

### Response to insects

Insects pose a particularly complex challenge to plants. Phloem-feeding, sucking insects typically cause limited tissue damage during feeding and induce the SA defence response. In contrast, chewing insects induce the JA/ABA co-regulated defence response, potentially inducing a similar (although not identical) defence response as necrotrophic pathogens due to the widespread tissue damage inflicted by both types of stressors. Rasping insects wound tissue prior to feeding on the exuding fluids, therefore causing chewing insect-like damage but using a similar feeding mechanism to sucking insects. Several species, including the chewing insect *Pieris rapae,* the phloem-feeder *Myzus persicae,* and the rasping insect *Frankliniella occidentalis,* are used as model organisms to investigate host-insect interactions in Arabidopsis.

Chewing insects, including *P. rapae*, typically induce the defence response co-regulated by JA and ABA. While several studies have found that the response to *P. rapae* is regulated by this defence network, the timing and pattern of defence signaling is somewhat variable. Coolen et al. (2016) characterized the global transcriptomic response to *P. rapae* infestation, noting that significant changes in gene expression were observed as early as 3 hpi and remained transcriptionally responsive at 24 hpi. A different timecourse was assayed by De Vos et al. (2005) who found the JA/ABA pathway marker *VEGETATIVE STORAGE PROTEIN 2* (*VSP2*) to be upregulated at 12 hpi before increasing in expression at 24 hpi and remaining upregulated at 48 hpi in response to insect attack (De Vos et al., 2005). In contrast, another study found that *P. rapae* infestation triggered a significant upregulation of *VSP2* at 24 hpi before declining in expression at 28 hpi and returning to basal levels of expression at 48 hpi (Vos et al., 2015). Interestingly, despite widespread damage, accumulation of JA gradually increased between 3 and 72 hpi but at relatively low levels (De Vos et al., 2005).

The sucking insect *M. persicae* induced the SA and JA defence networks. *M. persicae* feeding induced the expression of *PDF1.2* at 48 hpi, alongside the anti-fungal chitinase *PATHOGENESIS-RELATED 4* (*PR4*) at 48 and 72 hpi. Additionally, infestation with *M. persicae* also induced the SA marker *PR1* at the same time points (De Vos et al., 2005). This dual induction of the defence responses was also observed by Kerchev et al. (2013) wherein the SA markers, including PR1, were initially induced at 6 hpi before peaking at 24 hpi and declining in expression at 48 hpi. In the same study, the JA/ET marker genes, including VSP2, were stably upregulated at 6, 12, and 24 hpi.

Infestation with *F. occidentalis* and *M. persicae* triggered opposite expression patterns of the defence marker genes. Although both insects are classified as phloem-feeders, *F. occidentalis* is a rasping feeder whereas *M. persicae* is a stylet feeder, therefore presenting different challenges to the plant during infestation. The JA/ET pathway marker *PDF1.2*, typically associated with the response to necrotrophic pathogens, was upregulated in response to *F. occidentalis* at 12 and 24 hpi. This was followed by a gradual increase in the accumulation of JA between 12 and 72 hpi (De Vos et al., 2005). Several JA defence markers, including *PDF1.2* and *VSP2*, were upregulated in response to *F. occidentalis* – initially responding at 5 hpi before peaking at 10 hpi and beginning to decline at 24 hpi. Interestingly, two SA pathway markers, PR1 and the anti-microbial beta-1,3-glucanase 2 (BGL2), were also upregulated in response to *F. occidentalis* feeding, displaying increasing upregulation at 10 and 24 hpi (Abe et al., 2008). This is the opposite expression profile observed in response to *M. persicae* wherein the SA marker genes displayed a prolonged upregulation (6–48 hpi) and the JA markers were induced under a shortened expression profile (6–24 hpi; Kerchev et al., 2013).

In general, the response to insect attack was more variable than the response to microbial stressors. While the chewing insect *P. rapae* consistently induced the JA and ABA co-regulated defence pathway, the transcriptional dynamics in response to insect attack were variable. It is possible that the shifting timeline of defence induction and signaling observed across these studies was due to inconsistent timing and amount of tissue damage caused during larval feeding. Phloem-feeders concurrently induced the SA and JA/ET co-regulated defence responses. By inducing both defence networks, it is possible that inter-pathway signaling and crosstalk interfered with the typical defence signaling, therefore contributing to the variable expression profiles observed in response to *F. occidentalis* and *M. persicae*.

## The phytohormone networks display shared and unique pathway structures

**Figure 4:**
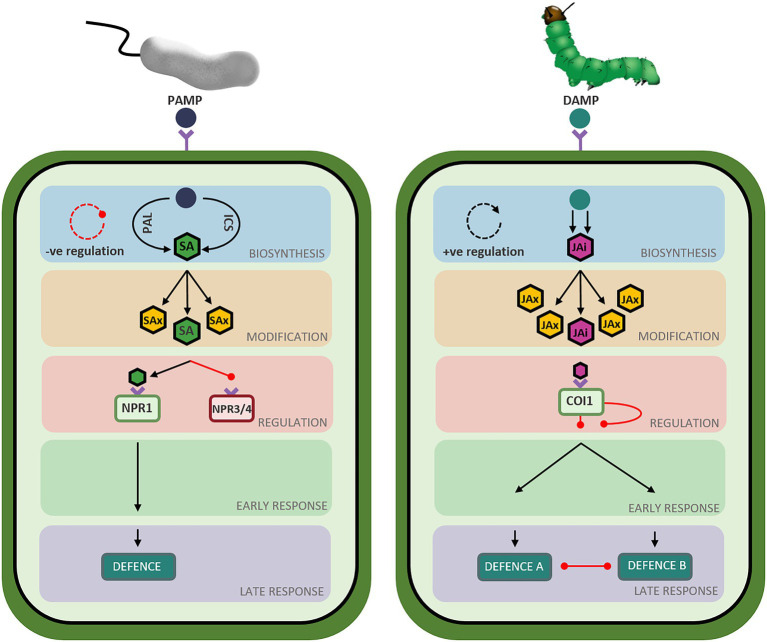
Overview of defence pathway structure in *Arabidopsis thaliana.* Schematic representation of the salicylic acid (left) and jasmonic acid (right) defence network stages in *Arabidopsis thaliana.* Triangle-ended black arrows represent activation of signaling and circle-ended red arrows represent inhibition of signaling. Left: Perception of Pathogen Associated Molecular Patterns (PAMPs) from infection with a biotrophic pathogen initiates the salicylic acid biosynthesis stage (blue-shaded box) composed of two short parallel pathways (ICS and PAL) resulting in the production of metabolically active salicylate (SA: green hexagon). A negative feedback loop regulates hormone biosynthesis, represented by the red circular arrow. Salicylate can be converted to various metabolically inactive forms (SAx: yellow hexagons) in the modification stage (orange-shaded box). Bioactive salicylate can bind to its receptors to activate NPR1 (green rectangle) and suppress NPR3 and NPR4 (red rectangle), relieving the repression of the signaling pathway. A signaling cascade begins in the early response stage (green-shaded box) and ends in the late response stage (purple-shaded box) resulting in the deployment of a defence response tailored to the perceived stressor. Right: Perception of Damage Associated Molecular Patterns (DAMPs) from insect attack initiates the jasmonic acid biosynthesis stage (blue-shaded box), composed of a long pathway resulting in the production of metabolically active jasmonic acid-isoleucine (JAi: pink hexagon). A positive feedback loop regulates hormone biosynthesis, represented by the black circular arrow. Jasmonic acid can be converted to various metabolically inactive forms (JAx: yellow hexagons) in the modification stage (orange-shaded box). Bioactive jasmonate isoleucine can bind to its receptor (green rectangle), relieving the repression of the signaling pathway. A signaling cascade begins in the early response stage (green-shaded box) and ends in the late response stage (purple-shaded box), resulting in the deployment of the jasmonic acid-mediated defence response best suited to countering the perceived stressor while simultaneously suppressing the other defence response (necrotroph versus herbivore).

While the SA and JA defence networks respond to different types of biotic stressors, both networks follow a similar set of ‘stages’ during defence induction. During the deployment of defence, the SA and JA signaling pathways transition through ‘stages’ of hormone biosynthesis, modification, perception, early response, and late response (Figure 4). While these stages are common between the two defence networks, the genetic components, regulatory mechanisms and structure of the signaling pathways are unique to each hormone (Figures 1, 2). Recent advancements in the understanding of the structure, components, and regulation of the SA and JA defence networks in the model plant Arabidopsis may offer clues as to why the SA and JA pathways display unique expression profiles.

### Phytohormone biosynthesis

#### SA biosynthesis is regulated by a negative feedback loop

In the SA network, shikimic acid is converted to SA (2-hydroxybenzoic acid) *via* two short pathways (Figure 1; Chen et al., 2009). While these pathways operate in a different set of cellular compartments, the biosynthetic routes finish in the cytosol, resulting in the production of bio-active SA in the same compartment as its receptor, NONEXPRESSOR OF PATHOGENESIS-RELATED PROTEIN (NPR1; see Section “SA perception and pathway activation is regulated by various post-translational modifications”). SA synthesis can occur *via* a phenylalanine intermediate in the phenylpropanoid (PAL) biosynthetic pathway, named after the four partially functionally redundant PAL genes (Klämbt, 1962; Huang et al., 2010). Alternatively, hormone production can also occur *via* an isochorismate intermediate in the isochorismate (ICS) pathway (Wildermuth et al., 2001; Garcion et al., 2008). In the ICS pathway, ISOCHORISMATE SYNTHASE 1 (ICS1) converts chorismate to isochorismate which is rapidly exported from chloroplasts into the cytosol by the exporter ENHANCED DISEASE SUSCEPTIBILIY 5 (EDS5) before undergoing further modification to produce bio-active SA (Wang et al., 2010; Serrano et al., 2013; Rekhter et al., 2019). Recently, an additional route that supplements SA signaling was identified wherein ENHANCED DISEASE RESISTANCE 1 (EDS1) and PHYTOALEXIN DEFICIENT 4 (PAD4) signal both through and independently of ICS1 to side-step the PAL and ICS pathways and bolster hormone accumulation and defence signaling (Cui et al., 2017; Hillmer et al., 2017; Rekhter et al., 2019; Torrens-Spence et al., 2019). SAR-Deficient 1 (SARD1) and Calmodulin Binding Protein 60 g (CBP60g) positively regulate the expression of SA biosynthetic genes ICS1, EDS5, and avrPphB SUSCEPTIBLE 3 (PBS3), resulting in the accumulation of SA and pathway activation following pathogen perception (Zhang et al., 2010; Wang et al., 2011; Sun et al., 2015).

Interestingly, several transcription factors negatively regulate SA biosynthesis. SA-responsive transcription factors, including WRKY70 and WRKY54, are rapidly upregulated following pathway induction. Once upregulated, these transcription factors directly bind to and inhibit SARD1 and CBP60g, resulting in the inactivation of SA biosynthetic genes and a reduction in hormone biosynthesis and accumulation (Zhou et al., 2018). Additionally, WRKY18 and WRKY40 directly bind and negatively regulate the biosynthetic genes ICS1, EDS5, and PBS3 (Birkenbihl et al., 2012). Collectively, the coordinated activity of several WRKY transcription factors act to dampen SA production and signaling following pathway activation, therefore generating a negative feedback loop wherein the induction of SA-induced transcription factors rapidly downregulate and limit further SA production (reviewed by Huang et al., 2020).

#### JA biosynthesis is regulated by a positive feedback loop

Two linear, biosynthetic routes control the production of JA (Figure 2). In the chloroplast, membrane galactolipids are cleaved by DEFECTIVE ANTHER DEHISCENCE 1 (DAD1) and other enzymes to release α-linolenic acid and hexadecatrienoic acid (Ishiguro et al., 2001). α-linolenic acid and hexadecatrienoic acid are then processed by a common suite of enzymes, including several LIPOXYGENASE (LOX) proteins, ALLENE OXIDE SYNTHASE (AOS) and ALLENE OXIDE CYCLASE (AOC; Stenzel et al., 2012; Gasperini et al., 2015; Chauvin et al., 2016) to form the mobile cyclopentenone 12-oxo-*cis*-10,15-phytodienoic acid (*cis*-OPDA) and dinor-12-oxo-phytodienoic acid (dnOPDA), respectively (Dave and Graham, 2012). *cis*-OPDA and dnOPDA then transit from the chloroplast into the peroxisome, mediated by the chloroplast outer envelope protein JASSY and the peroxisomal transporter COMATOSE1 (CTS1; Theodoulou et al., 2005; Guan et al., 2019), where they are reduced by OPDA REDUCTASE 3 (OPR3) to produce the metabolically inactive, backbone molecule 7-*iso*-jasmonic acid (hereafter referred to as 7-*iso*-JA; Stintzi and Browse, 2000). Alternatively, OPR3-independent 7-*iso*-JA synthesis can occur in the cytosol wherein the reduction of *cis*-OPDA and dnOPDA is catalyzed by OPDA REDUCTASE 2 (OPR2) instead (Chini et al., 2018). Interestingly, while several signaling roles have been described for *cis*-OPDA, hexadecatrienoic acid-derived dnOPDA has not been associated with distinct function(s) in Arabidopsis (Dave and Graham, 2012).

To activate signaling, 7-*iso*-JA must be conjugated to the amino acids alanine, valine, methionine, or leucine, with each conjugate varying in bioactivity and role *in planta* (Yan et al., 2016). Despite the multitude of jasmonates present *in planta*, two metabolically active forms have been identified as the main drivers of JA defence signaling; 7-*iso*-jasmonoyl-L-isoleucine (JA-Ile) and its derivative 12-hydroxy-jasmonoyl-isoleucine (12-OH-JA-Ile). Interestingly, while both jasmonates have overlapping roles *in planta*, 12-OH-JA-Ile appears to be particularly involved in the response to wounding (Jimenez-Aleman et al., 2019; Poudel et al., 2019). The jasmonate-amido synthetase JASMONATE RESISTANT 1 (JAR1) conjugates an isoleucine group to 7-*iso*-JA to form JA-Ile (Staswick and Tiryaki, 2004; Suza and Staswick, 2008) which can be further hydroxylated by the cytochrome P450, CYP94C1, to produce 12OH-JA-Ile (Heitz et al., 2012, 2016; Koo et al., 2014). These modifications are rapid, with JA and JA-Ile accumulating within minutes following pathway induction (Glauser et al., 2009). Bioactive JA-Ile and 12-OH-JA-Ile can then bind to their receptor, CORONATINE INSENSITIVE 1 (COI1), and initiate signaling (see Section “Comparison of the regulation and perception stage of the SA and JA defence networks”).

Key JA biosynthetic genes, including *LOX, AOS, AOC, OPR3* and *JAR1* are upregulated in response to JA. These genes are positively regulated by a JA-responsive basic helix–loop–helix leucine zipper (bHLH) transcription factor, MYC2 (also known as JIN1 or JAI1). JA-Ile accumulation then triggers further production of MYC2 to bolster additional phytohormone synthesis, therefore generating a positive feedback loop wherein the biosynthesis of JA promotes further hormone synthesis (previously reviewed by Wasternack, 2007; Browse, 2009; Wasternack and Hause, 2013). Conversely, hormone accumulation also triggers the induction of negative regulators of the JA pathway (JASMONATE ZIM-domain (JAZ) proteins), however these repressors acts downstream at the perception stage (see Section “Comparison of the regulation and perception stage of the SA and JA defence networks”) of the defence networks (Chung et al., 2008).

#### Comparison of the biosynthetic stage of the SA and JA defence networks

The biosynthetic stage has evolved unique structures in the SA and JA defence networks (Figure 4). In the SA network, biosynthesis occurs *via* two short pathways to produce metabolically active SA. Owing to this pathway structure, newly synthesized bioactive salicylate accumulates co-localized with its receptor in the cytosol, facilitating the immediate induction of defence signaling. In contrast, two long, spatially segregated pathways control biosynthesis in the JA network to produce jasmonic acid. Additionally, the phytohormone networks are subjected to contrasting regulatory mechanisms. SA synthesis and accumulation is controlled by a negative feedback loop wherein hormone production activates the expression of a subset of WRKY transcription factors tasked with downregulating further hormone synthesis and accumulation. In contrast, the JA biosynthetic route is controlled by a positive feedback loop wherein biosynthesis of JA activates the upregulation of biosynthetic and regulatory genes to promote further production and accumulation of JA upon pathway activation. The observed structural and regulatory differences between the SA and JA biosynthetic pathways may, in part, contribute to the contrasting expression profiles of the defence hormone networks.

### Modification of bioactive phytohormones

Hormones exist in various forms *in planta*, allowing for the rapid shuttling of precursors and derivatives in and out of active ‘circulation’. Chemical modification is an important regulatory stage within the defence networks whereby the molecular structure, membrane permeability, activity, compartmentalization, and function of the hormone can be altered. Hormone accumulation at the site of infection has been a known prerequisite for defence induction since the 1990’s (Metraux et al., 1990; Doares et al., 1995), however it has remained unclear whether defence signaling is predominantly activated by newly synthesized, or re-activated storage forms of the phytohormones. More recent research highlighting the large pools of hormone pre-requisites in uninfected tissue, alongside the speed of defence induction relative to hormone biosynthesis, suggest that the latter is more likely (Mou et al., 2003; Browse, 2009).

#### Modification as a means of pathway suppression in the SA defence network

Newly synthesized SA is metabolically active. Modifications, including glycosylation, hydroxylation, and methylation, convert SA to derivatives with varying degrees of bioactivity (Figure 1; Lefevere et al., 2020). During glycosylation, SA GLUCOSIDE TRANSFERASE 1 (SAGT1) and SAGT2 conjugate a glycosyl group to SA, rapidly converting metabolically active SA to an inactive glucoside-conjugate. These derivatives are then stored in the vacuole until required by the plant (Dean and Delaney, 2008; Song et al., 2009; Vaca et al., 2017). Alternatively, glycosylation can dampen defence signaling. Recent studies have identified the role of UDP-DEPENDENT GLYCOSYLTRANSFERASE 76B1 (UGT76B1) in the glycosylation and subsequent inactivation of SA and N-hydroxy-pipecolic acid (NHP); the mobile signal for systemic acquired resistance (Bauer et al., 2021; Holmes et al., 2021; Mohnike et al., 2021). Additional modifications, including methylation catalyzed by BA/SA CARBOXYL METHYLTRANSFERASE 1 (BSMT1; Chen et al., 2003; Song et al., 2009), and hydroxylation, catalyzed by DOWNY MILDEW RESISTANT 6 (DMR6; Zhang et al., 2017) and DMR6-LIKE OXYGENASE 1 (DLO1; Zeilmaker et al., 2015), generate inactive forms of SA. Collectively, these modifications convert metabolically active SA into various inactive derivatives, rapidly disabling and/or dampening the SA signaling response and facilitating return to basal hormone levels. Interestingly, inactive MeSA can be re-activated by the methyl esterase activity of several several *MES* (methyl esterase) genes, allowing the plant to switch between inactive MeSA and active SA as required (Vlot et al., 2008).

#### Modification facilitates dynamic activation and de-activation of the JA defence network

The final steps of the JA biosynthetic pathway involve the modification of the inactive 7-*iso*-JA into bioactive JA-Ile and/or 12-OH-JA-Ile. JA-Ile and 12-OH-JA-Ile are subjected to complex enzymatic regulation with two independent pathways catalyzing hormone turnover and deactivation of JA signaling; oxidation, catalyzed by CYP94B3/1 and CYP94C1 (Koo et al., 2011; Heitz et al., 2012; Bruckhoff et al., 2016), and amidohydroxylation back to 7-*iso*-JA catalyzed by IAR3 and ILL6 (Widemann et al., 2013). Conversely, other modifications to the 7-*iso*-JA can occur in the cytosol, resulting in the production of jasmonates with varying roles and activity *in planta*. Like SA, 7-*iso*-JA can be methylated or hydroxylated to generate inactive derivatives, catalyzed by JASMONATE-METHYL TRANSFERASE 1 (JMT1) and JASMONATE-INDUCED OXYGENASE (JOX), respectively (Seo et al., 2001; Caarls et al., 2017). Similarly, removal of the methyl group, catalyzed by MeJA-specific Methyl Esterase (MJE), returns MeJA to the backbone 7-*iso*-JA (Koo et al., 2013). Collectively, enzymatic modification results in the production of various JA derivatives (Figure 2). Interestingly, with the exception of the JOX genes, most negative modifications appear to be reversible and coupled with a positive modification (as demonstrated by the activity of JAR1 and IAR3/ILL6, and JMT1 and MJE), facilitating the dynamic cycling of jasmonates between active and inactive forms, as required by the plant.

#### Comparison of the modification stage of the SA and JA defence networks

Hormone modification allows the plant to rapidly switch between active and inactive states of the defence response. In the SA network, most modifications to the newly synthesized hormone act to convert metabolically active SA to inactive or storage forms, including those catalyzed by DMR6, DLO1, BSMT1, SAGT1/2, and UGT76B1. As such, the structure of the modification stage facilitates rapid disabling or dampening of defence signaling, avoiding over-activation and costs associated with the SA defence response. One of such costs is the hypersensitive response: a defence mechanism whereby pathogen recognition triggers programmed cell death to limit pathogen spread at the expense of host tissue. In the JA network, the metabolically inactive 7-*iso*-jasmonate backbone must be converted JA-Ile or 12-OH-JA-Ile to activate defence, but can also be modified into jasmonates with varying activities, mobility, and roles *in planta*. Many enzymatic modifications produce inactive JA (IAR3, ILL6, JOX, JMT, CYP94C1), however, they are often paired with an enzyme that reverts inactive JA to bioactive JA, or the backbone 7-*iso*-jasmonate (JAR1, MJE, CYP94B3/1). Transport of the hormone and its derivatives may add an additional layer of regulation to defence induction. Modification of SA and JA can then further alter the mobility of the hormone, facilitating movement within and between plant tissues to alter the rate of defence induction (thoroughly reviewed by Nguyen et al. (2017) and Kachroo et al. (2020)). While there does not appear to be a clear explanation for the difference in the SA and JA expression profiles at this stage of the defence networks, it is possible that the sheer number of deactivating opportunities may lead to a faster turning off of the SA pathway, compared to JA (Figure 4).

### Phytohormone perception and initiation of defence signaling

Metabolically active hormones must bind to their receptors to initiate defence signaling. Post-translation modifications of the hormone receptors play a significant role in the regulation of defence. However, the mechanisms and modifications deployed in the SA and JA defence networks differ.

#### SA perception and pathway activation is regulated by various post-translational modifications

##### Suppression of SA defence in unstressed plants

The NONEXPRESSOR OF PATHOGENESIS-RELATED PROTEIN (NPR) protein complex controls pathway activation in the SA defence network (Figure 1). NPR1 (also called NIM1), and homologs NPR2, NPR3 and NPR4 are SA receptors located in the cytosol (Kinkema et al., 2000). In the unstressed state, SA binds to NPR3 and NPR4 which, alongside their signaling partners TGA2/5/6, repress the expression of downstream defence genes, including WRKY transcription factor 70 (see Section “SA early response”; Ding et al., 2018). It was previously proposed that intermolecular di-sulfide bonds bind NPR1 into an oligomeric complex effectively trapping the receptor in the cytosol (Mou et al., 2003). Additionally, NIM1-INTERACTING PROTEIN 3 (NIMIN3) binds to the N-terminus of NPR1 to constitutively repress its activity in unstressed plants (Weigel et al., 2001; Hermann et al., 2013). The cellular localization of this protein–protein interaction remains unclear, but likely occurs in the nucleus, along with the other NIMIN proteins. Interestingly, alternative SA receptors functioning independently of NPR1 have also been identified. These SA-binding proteins (SABPs) have varying, often undefined roles, but add to the complexity of the SA defence network, thoroughly reviewed by Pokotylo et al. (2019). Of these, CATALASE2 (CAT2) is of particular interest due to its proposed role as a SA-activated antagonist of the auxin and JA signaling networks (Yuan et al., 2017).

##### Regulation of SA defence in stressed plants

Stress perception triggered accumulation of SA has contrasting effects on the NPR receptors (Figure 1). High levels of SA inhibit the co-repressor activity of NPR3 and NPR4, thus releasing their repression of the SA pathway (Ding et al., 2018). Concurrently, SA binds to NPR1 oligomers. Once a threshold level of SA has bound, thioredoxin H-type 3 (TRX-h3) and thioredoxin H-type 5 (TRX-h5) will catalyze the *S-*nitrosylation and subsequent reduction of oligomeric NPR1. The resultant monomeric NPR1 then transits from the cytosol into the nucleus (Kinkema et al., 2000; Mou et al., 2003; Tada et al., 2008). However it is important to note that the oligomer-monomer transit model has been questioned by the recent finding that NPR1 predominantly exists in its monomeric form *in vivo*, regardless of the intracellular levels of SA, and that the oligomerisation may be due to *in vitro* conditions (Ishihama et al., 2021).

Once in the nucleus, NPR1 activity is regulated by a number of proteins and post-translational modifications. Upon SA accumulation, NIMIN2 is rapidly upregulated and binds to the C-terminus of NPR1, displacing the NIMIN3 repressor and bolstering NPR1-mediated signaling induction. NIMIN1, displaying delayed induction by SA, binds to the C-terminal of NPR1 to displace NIMIN2 and transiently activates the expression of genes involved in the later response to SA, effectively ‘activating’ the NPR1 receptor (Weigel et al., 2001; Hermann et al., 2013). Concurrently, conjugation of Small Ubiquitin-like Modifier (SUMO) proteins to NPR1 causes the receptor to convert its association with transcription repressors to transcriptional activators. This modification is inhibited by phosphorylation, facilitating complex and fine-tuned control of NPR1 activity (Saleh et al., 2015). ‘Active’ NPR1 can associate with transcriptional co-activators, including TGA2/5/6, EDS1, and the histone acetyltransferases HAC1 and HAC5, to activate the transcription of downstream SA responsive genes (Zhang et al., 2003; Jin et al., 2018; Chen et al., 2021). Additionally, the NPR1-mediated activation of the SA pathway is coupled with the antagonism of the JA pathway. At the promoter region of JA-responsive genes, NPR1 associates with MYC2 and blocks the binding of the co-activator MEDIATOR 25 (MED25), repressing gene transcription and defence signaling (Nomoto et al., 2021). Recently, Castelló et al. (2018) found that NPR2 interacts and shares partial function with NPR1, therefore highlighting the potential of this paralogue to act as an additional, functionally redundant SA receptor.

Removal of ‘spent’ NPR1 from the promoters of target genes is regulated by polyubiquitination and proteasomal degradation. Upon entering the nucleus, NPR1 is phosphorylated and ubiquitinated by a Cullin-RING E3 ubiquitin ligase CRL3 (Spoel et al., 2009). Initially, this modification increases NPR1 activity, further bolstering the activation of downstream genes. As gene activation continues, NPR1 is ligated to additional ubiquitin tags by the E4 ubiquitin ligase UBE4, resulting in polyubiquitination and consequent proteasomal degradation. This turnover of ‘used’ NPR1 allows any remaining active NPR1 monomers to continue gene activation. The activity of two proteasome-associated deubiquitinases, UBP6/7, remove the ubiquitin tags to prolong NPR1 longevity and gene activation, therefore fine-tuning defence signaling (Skelly et al., 2019).

#### The JA defence network is regulated by a major repressor complex

##### Suppression of JA defence in unstressed plants

The JA defence network is controlled by a major regulatory hub operating at perception of bioactive JA-Ile (Figure 2). In the unstressed state, the JA defence response is constitutively repressed by 13 partially functionally redundant JASMONATE ZIM DOMAIN (JAZ) repressor proteins (Chini et al., 2007; Thines et al., 2007; Yan et al., 2007; Guo et al., 2018). The JAZ proteins recruit the co-repressors NINJA, TOPLESS (TPL) and TOPLESS-LIKE REPRESSORs (TPRs) to form a multi-protein complex which binds to the promoters of JA responsive genes and blocks their transcription (Chini et al., 2007; Thines et al., 2007; Yan et al., 2007; Pauwels et al., 2010; Hickman et al., 2017). SUMOylation of the JAZ proteins act to stabilize the JAZ repressors and inhibit binding of the COI1 receptor (Srivastava et al., 2018). Interestingly, different overlapping subsets of the JAZ proteins appear to repress the two branches of the JA defence response. JAZ1, 2, 3, 5, 6, 8, 9, 10, 11, 12, and 13 act to constitutively repress the MYC branch of the JA defence response (Fernández-Calvo et al., 2011), whereas this role is held by JAZ1, 3, and 9 in the ERF defence response (Zhu et al., 2011).

##### Regulation of JA defence in stressed plants

Stressor perception induces the expression of the SUMO proteases OVERLY TOLERANT TO SALT1 (OTS1) and OTS2 which remove the SUMO proteins from the JAZ repressors, reducing their stability and allowing binding of the receptor (Figure 2; Srivastava et al., 2018). COI1 can then form a co-receptor with JAZ proteins to bind JA-Ile. As a member of the SCP-Cullin-F-box (SCF) ubiquitin ligase complex (Yan et al., 2009; Sheard et al., 2010), COI1 tags the JAZ proteins for degradation in the 26S proteasome (Thines et al., 2007; Katsir et al., 2008; Ye et al., 2012), resulting in the disassembly of the JAZ-NINJA-TPL-TPR repressor complex (Pauwels et al., 2010). Interestingly, the SA receptors NPR3 and NPR4 also interact with, and promote the reduction of, intracellular JAZ1 (Liu et al., 2016). Repressor degradation removes the inhibition of downstream JA-responsive genes to allow pathway activation. Transcription factors also aid in the regulation of defence signaling. Targets of the JAZ repressor protein themselves, a suite of bHLH subgroup IIId transcription factors (bHLH3/13/14/17) function redundantly to repress JA signaling *via* direct binding and antagonizing JA transcriptional activators including MYC2 (Song et al., 2013).

The mechanism behind the restoration of JAZ-mediated pathway suppression is likely to be dependent on the abundance of intracellular JA-Ile. While the JAZ repressor complex is rapidly degraded following pathway activation and the perception of JA-Ile, JA signaling also triggers the upregulation of JAZ gene expression within 1 h post pathway induction (Thines et al., 2007; Chung et al., 2008). Given this rapid replenishment of repressor proteins, it is unclear why newly synthesized JAZ proteins do not immediately suppress the JA pathway. However, as the JAZ/COI1 protein complex (rather than COI1 alone) are required for the perception of JA-Ile and 12OH-JA-Ile, JAZ proteins must be available in order to bind to COI1 and facilitate JA signaling. This, coupled to the positive feedback looping acting on JA biosynthesis, would ensure continual production of the JAZ proteins to allow the establishment of JA signaling, perhaps also explaining the prolonged expression profile observed in the JA pathway. In the absence of JA-Ile, the JAZ repressor proteins would not be bound by COI1 allowing pathway repression to be restored.

#### Comparison of the regulation and perception stage of the SA and JA defence networks

Acting as a gatekeeper to defence activation, binding of the hormone to its receptor(s) links the perception of stress to the activation of defence. While various transcriptional, post-translational, and epigenetic modifications control the perception and activation of the defence networks, the SA and JA networks rely on contrasting regulatory mechanisms to initiate defence signaling (Figure 4). SA perception and defence activation is initially regulated by a threshold, switching from NPR3- and NPR4-mediated pathway repression to pathway activation by NPR1, triggered by hormone accumulation. Once in the nucleus, NPR1 is regulated by a number of concurrent post-translational modifications and can only activate defence signaling after associating with co-activators. As such, the activation of NPR1 and SA-mediate defence is tightly regulated by various mechanisms acting in concert to allow the fine-tuning of defence.

In contrast, the JA defence response relies on a major regulatory complex acting at the COI1 receptor to control pathway activation. The JA defence network is constitutively repressed by the activity of the JAZ repressor proteins. Pathway activation can only occur once the COI-JAZ repressor complex is bound by JA-Ile and the JAZ repressor complex is degraded in the 26S proteasome. Additional factors aid in the control of the JA pathway, however the JA defence network appears to have relatively fewer regulatory steps than the SA defence response. Interestingly, different JAZ protein compliments were found to repress the MYC and ERF branches of the defence response, highlighting the potential for neosubfunctionalisation of these repressor proteins having aided in the delineation of the two pathways (Fernández-Calvo et al., 2011; Zhu et al., 2011).

### Early response

The early response stage, driven by the activity of transcription factors, links hormone perception to defence induction. Often targets of the hormone receptors and their co-activator proteins themselves, transcription factors display altered expression early in the defence response. These transcription factor proteins do not have a direct role in defence, but instead regulate the expression of downstream genes. Hickman et al. (2017) defined the early response genes as those showing altered expression patterns within 15 min to 2 h following pathway induction.

#### SA early response

WRKY and TGA transcription factors dominate in the early response stage in the SA network (Hickman et al., 2019). Several transcription factors, including WRKY70 and its homologue WRKY54, have been found to promote the activation of defence signaling by inducing the expression of late response genes, including PATHOGENESIS-RELATED 1 (PR1) and PR2 (Figure 1; Li et al., 2004). Additionally, WRKY70 and WRKY54 repress genes within the JA signaling network, highlighting their role as key crosstalk nodes (Li et al., 2006, 2017). Negative regulators of the SA defence response are also upregulated early in defence signaling. Similar to the modification stage, the early response stage of the SA network rapidly induces the expression of genes involved in the promotion of the SA defence response, while simultaneously inducing the expression of negative regulators of the SA defence response to establish a negative feedback loop to limit hormone biosynthesis and further defence signaling (Ding et al., 2018; Zhou et al., 2018; Hickman et al., 2019).

#### JA early response

The JA defence network is tasked with responding to a diverse range of attackers, ranging from tortoises, to caterpillars and fungal pathogens (De Vos et al., 2005; Verhage et al., 2011; Mafli et al., 2012). The JA defence response is delineated into two pathways, each tailored to a specific type of defence (Figure 2; Lorenzo et al., 2004). The MYC-branch of the JA defence response controls defence against chewing insects, herbivory, and wounding and is co-regulated by abscisic acid. Centered on its namesake, the MYC-branch is regulated by MYC2. Upon wounding or herbivory, co-regulator MED25 associates with MYC2 and its homologs MYC3 and MYC4 to initiate downstream signaling (Lorenzo et al., 2004; Çevik et al., 2012; Vos et al., 2013). Necrotrophic pathogens induce the second, ET co-regulated, ERF-branch of the JA defence response, centered on the transcription factor ETHYLENE RESPONSE FACTOR 1 (ERF1). In unstressed plants, ETHYLENE-INSENSITIVE 3 (EIN3) associates with a histone deacetylase, HDA6 to repress defence gene expression (Zhu et al., 2011). Pathogen perception triggers the production of JA and ET, causing disassembly of HDA6 and EIN3 (De Vos et al., 2005; Zhu et al., 2011). Freed from HDA6, EIN3 and its signaling partners EIN3-LIKE1 and 2 (EIL1 and EIL2) upregulate the expression of ERF1 (Dolgikh et al., 2019). ERF1 and its functionally redundant signaling partner OCTADECANOID-RESPONSIVE ARABIDOPSIS AP2/ERF 59 (ORA59) then alter the expression of downstream genes to activate defence (Lorenzo et al., 2003; Pre et al., 2008).

#### Comparison of the early response stage of the SA and JA pathways

The early response stage of the defence networks is tasked with the initiation of defence signaling. The SA early response stage is shaped by a defined group of transcription factors, all of which rapidly respond to pathway induction and direct the deployment of a universal SA defence response. In contrast, the JA defence response consists of two, mutually antagonistic transcription factor hubs which function to tailor the defence response to the perceived stressor (Figure 4). Unlike SA, the JA early response stage is heavily regulated by inter-pathway communication. The MYC and ERF branches are co-regulated by ABA and ET, respectively, therefore requiring synergistic signaling between these hormone networks to initiate defence deployment (Anderson et al., 2004; Pre et al., 2008; Zhu et al., 2011). Additionally, the MYC and ERF branches are mutually antagonistic, requiring some of the plant’s resources and energy to be diverted to repress the opposing pathway during defence activation (Lorenzo et al., 2003). Collectively, the structure of the SA early response facilitates unidirectional induction of defence, whereas the JA pathway is branched, potentially providing a more diverse defence repertoire to allow the response to a broader range of biotic stressors.

### Late response

The late response stage of the defence networks is composed of a large group of genes displaying altered expression 4 h or more post-pathway induction. These genes are the end-point of the signaling cascade and execute various roles *in planta*. Late response genes are often selected as “reporter genes” because of their significant and prolonged upregulation following pathway activation. The most commonly used reporter genes for studies in Arabidopsis are detailed here.

#### SA late response

PR1 and PR2 are widely used markers of the SA defence response. Induced by WRKY70, PR1 and PR2 expression is significantly upregulated following infection with biotrophic pathogens (Figure 1). PR1 has reported sterol-binding antimicrobial properties useful in defence against insects and pathogens unable to synthesize their own sterol (Gamir et al., 2017; Jing and Behmer, 2020), while PR2 is a β-1,3-glucanase with antifungal properties (Balasubramanian et al., 2012).

#### JA late response

Like their early response counterparts, the late response genes are segregated into two branches to facilitate attacker-specific defence induction in the JA signaling network (Figure 2; Lorenzo et al., 2004). In the MYC branch, MYC2 activates a subset of NAC transcription factor family genes, namely NAC019, NAC055 and NAC072, which induce the upregulation of the VEGETATIVE STORAGE PROTEIN (VSP) proteins (Bu et al., 2008; Zheng et al., 2012; Cui et al., 2018). VSP1 and VSP2 are acid phosphatases which display increased expression following tissue wounding (Berger et al., 2002; Chen et al., 2012). Although their mechanism of action remains unclear, the phosphatase activity of VSP2 has been linked to delayed development and increased mortality in feeding insects (Liu et al., 2005).The plant defensin family, encoding 13 putative members, is a group of structurally related proteins with broad anti-fungal activity (Thomma et al., 2002). PLANT DEFENSIN 1.2 (PDF1.2) is regulated by ERF1 and ORA59 and induced in response to necrotrophic pathogens (Manners et al., 1998). While the mode of action of PDF1.2 remains elusive, this gene is regularly used as a marker for the ERF-branch of the JA defence network.

#### Comparison of the late response stage of the SA and JA defence networks

While reporter genes highlight the segregation of the SA and JA defence networks (Figure 4), they account for only a small number of genes within the late response stage. Unlike previous stages, the late response stages of the SA and JA defence networks converge, as evidenced by the advent of next generation sequencing transcriptomic studies which have highlighted the overlaps in transcriptional expression profiles following activation of the SA and JA pathways (Hickman et al., 2017, 2019). Here, synergistic and additive inter-pathway communication appears to coordinate the deployment of a general stress response, activated by either of the defence networks, with a comparatively smaller proportion of genes acting exclusively in a single pathway (reviewed by Aerts et al., 2021). Interestingly, both biotic and abiotic stressors induce this general defence response, with a third of differentially expressed genes showing similar expression patterns in response to opposing challenges (Coolen et al., 2016).

## Phytohormone defence networks may be shaped by multiple factors

Since the evolution of the earliest land plants ~470 million years ago, plants have been subjected to various biotic and abiotic stressors (Rubinstein et al., 2010). The defence phytohormone networks have evolved to allow the plant to respond to internal and external cues within the environment. These networks have undergone significant changes over time, as evidenced in the divergence of the signaling pathways along the lineage of land plants (Monte et al., 2018). It is likely that many factors have shaped the evolution of the defence networks.

### Phytohormone transcriptomic profiles may be influenced by pathway structure

The SA and JA defence responses display contrasting transcriptomic profiles in response to pathway induction (Figures 3, 4). The response to SA and biotrophic stressors trigger a rapid, dynamic, and transient SA-mediated defence response (Hickman et al., 2019; Zhang et al., 2020). In contrast, the JA defence response is more prolonged (Hickman et al., 2017; Zhang et al., 2020). While many factors contribute to the temporal profile of the defence responses, pathway structure likely plays a significant role in determining the transcriptomic profile of the defence networks.

Each stage of the SA signaling pathway is highly regulated to facilitate the fine-tuning of defence (Figure 1). Biosynthesis of the activate hormone is regulated by a negative feedback loop, facilitating rapid deactivation of defence signaling at the initiation of the pathway (Birkenbihl et al., 2012; Huang et al., 2020). Following biosynthesis, several SA derivatives can be formed, altering the flux of defence signaling from an active to an inactive state (Vlot et al., 2008; Song et al., 2009; Zeilmaker et al., 2015; Vaca et al., 2017; Zhang et al., 2017; Bauer et al., 2021; Holmes et al., 2021; Mohnike et al., 2021). Bioactive SA can bind to its receptor, NPR1, initiating the perception stage of the defence network. Here, NPR1 and co-receptors are subjected to several transcriptional and post-translational regulatory mechanisms to facilitate the tightly controlled and highly coordinated deployment of defence (Kinkema et al., 2000; Weigel et al., 2001; Mou et al., 2003; Zhang et al., 2003; Tada et al., 2008; Spoel et al., 2009; Hermann et al., 2013; Saleh et al., 2015; Castelló et al., 2018; Ding et al., 2018; Jin et al., 2018; Skelly et al., 2019; Chen et al., 2021). Defence deployment follows a relatively unidirectional signaling pathway in the early response stage, acting to simultaneously activate downstream signaling while simultaneously establishing an early and robust negative feedback loop to counter prolonged pathway induction (Ding et al., 2018; Zhou et al., 2018; Hickman et al., 2019).

By comparing the transcriptomic profile in the response to SA or biotroph infection, a general trend can be observed (Hickman et al., 2019; Zhang et al., 2020). The SA defence network initiates rapidly post-induction, dynamically modifying the expression of a large number of genes before returning to near basal expression levels within the assayed time courses (Figure 3). This rapid, dynamic, and transient transcriptomic profile may be explained by the structure of the SA pathway. The regulatory steps of each stage of the SA network act to inhibit or modulate the progression to the next stage, effectively limiting and/or deactivating defence signaling. Acting in concert, these regulatory mechanisms fine-tune the progression of defence signaling, potentially facilitating the observed transcriptomic profile.

Similarly, the transcriptomic profile observed in the JA signaling pathway(s) may be explained by the structure of its defence network (Figure 2). Production of bioactive JA is controlled by a positive feedback loop, wherein biosynthesis triggers further hormone accumulation and defence induction (Wasternack, 2007; Browse, 2009; Wasternack and Hause, 2013). Downstream of biosynthesis, the hormone modification stage results in the production of jasmonates with varying bioactivity and roles *in planta*. Many of these modifications can be converted back to the backbone state, allowing dynamic switching between active and inactive states of defence signaling as required by the plant (Seo et al., 2001; Koo et al., 2011; Heitz et al., 2012; Widemann et al., 2013; Bruckhoff et al., 2016; Caarls et al., 2017). The JA network is controlled by a central regulatory hub acting as a ‘gatekeeper’ to defence induction. Proteasomal degradation of the JAZ repressor proteins release the constitutive repression of the defence response, allowing rapid induction of downstream signaling (Thines et al., 2007; Katsir et al., 2008; Yan et al., 2009; Pauwels et al., 2010; Sheard et al., 2010; Ye et al., 2012). Once activated, the branched structure of the early response stage facilitates the rapid response to a broad range of biotic and abiotic stressors (Lorenzo et al., 2004).

The JA defence network operates a rapid, but prolonged reprogramming of the plant transcriptome in response to hormone treatment or biotic stressors (Figure 3; Hickman et al., 2017; Zhang et al., 2020). Like the SA network, the regulatory mechanisms and pathway structure of the JA defence network may contribute to the observed temporal profile. The JA biosynthetic pathways are largely controlled by positive feedback loops and the activity of a central regulator hub: the JAZ repressor proteins. Once activated, the structure and regulatory mechanisms of the JA pathway facilitate further pathway activation, bolstering hormone accumulation and downstream signaling. Together, these factors may explain the prolonged transcriptomic profile observed in response to pathway induction.

The reliance of both defence networks on proteasomal degradation as a method of pathway regulation may further contribute to the observed differences in the temporal profile of defence (Figures 1, 2). During defence signaling, the SA receptor NPR1 and the JA JAZ repressor proteins are degraded, therefore reducing protein availability. Defence signaling has been linked to a reduction in protein synthesis, although it remains unclear whether this is a global or more targeted reduction (Hickman et al., 2017, 2019). If global, the reduction of NPR1 synthesis would result in the dampening of the SA mediated defence response, due to the inability to induce the expression of downstream genes. In contrast, reduced production of the JAZ proteins under a global protein synthesis reduction model would remove pathway repression and promote JA defence signaling. Collectively, the impact of proteasomal degradation of these key regulatory genes may further contribute to the establishment of a rapid and transient SA defence response, and the prolonged JA defence response.

It is important to note that the structure and components of the SA and JA defence networks are not fully understood and new components are still being discovered. Future studies will aid in the elucidation of the defence networks and may provide additional insight into how pathway structure and defence deployment are linked.

### Pathway structure may have evolved to match the stressor

Biotic stressors may have contributed to the shaping of the defence networks. Biotrophic pathogens feed on living host cells often causing small-scale, temporally limited, and localized tissue damage, albeit with some exceptions. The SA defence network, tasked with responding to biotrophic pathogens, displays a rapid, dynamic, and transient response to counter this type of attack (Figure 3; Hickman et al., 2019; Zhang et al., 2020), often resulting in the hypersensitive response to restrict pathogen spread. While difficult to prove experimentally, it is tempting to hypothesize that this spatially- and temporally-limited, minimal damage, and rapid infection profile of the biotrophic pathogens may have shaped the evolution of the ‘short and sharp’ SA defence response.

Similarly, the structure and temporal profile of the JA defence network may have evolved to mimic the infection patterns of necrotrophic pathogens. These pathogens feed on dead host tissue, typically causing destructive tissue damage to host plants. It is possible that this significant stress signal is linked to the transcriptomic profile of the JA defence response wherein signaling is rapidly induced and remains stably upregulated for an extended timecourse (Figure 3). Of course, insect attackers also induce the JA defence response, but the temporal response to this type of stress appears to be more variable. Insect attack causes large-scale, often temporally and spatially diverse, destructive tissue damage, potentially accounting for the variability in defence profiling. Additionally, once the immediate stressor has been mitigated, necrotroph and/or insect damaged tissue is vulnerable to secondary attack by opportunistic necrotrophic pathogens, and so the JA defence network may remain upregulated for longer to protect the plant during tissue repair.

Plants are often subjected to concurrent or sequential challenge(s) by biotic and abiotic stressors in the environment. Under sequential attack, Arabidopsis rapidly shifts its defence response to counter the secondary stressor, while retaining the ‘signature’ response to the initial stressor (Coolen et al., 2016). The timing and type of defence signaling deployed in response to stress are likely to be dynamic, adapting to the immediate needs of the plant and the current challenges within the environment.

### Diverse roles *in planta* may contribute to pathway structure

Phytohormones typically have various roles *in planta*, which may have contributed to the evolution of the structure and regulation of the defence pathways. While SA contributes to the regulation of other aspects of plant function, its main task is the response to stress (Dempsey and Klessig, 2017; Koo et al., 2020). By evolving a rapidly inducible, highly-regulated, and transient pathway structure, the SA defence network can execute its defence response requirements while avoiding the negative consequences of pathway activation, including over-activation of the hypersensitive response and unnecessary antagonism of opposing hormone networks Koornneef et al. (2008). In contrast, JA regulates various growth and developmental roles, alongside its stress responsive roles *in planta*, thoroughly reviewed by Huang et al. (2017). As such, the JA network must be capable of facilitating hormone production and signaling during stressed and non-stressed states, therefore reducing the requirement for rapid pathway activation and deactivation in favor of a prolonged defence response. Again, these hypotheses would be difficult to test experimentally.

### Pathogen-mediated selection pressure may be linked to protein diversification

Targets of pathogen manipulation may undergo diversification and neofunctionalisation, further shaping the structure and transcriptomic profiles of the defence responses. During infection, pathogen effectors can disable, modulate, or inactive host defence genes to promote virulence. To maintain protein diversity and avoid effector-mediated defence disabling, certain genes may have undergone diversification and neofunctionalisation and/or epigenetic changes, as seen in the diversification of *NPR1, PAD4,* and *EDS1* and other defence-associated genes (Caldwell and Michelmore, 2009; Faillace et al., 2019; Hannan Parker et al., 2022). The diversification of particular proteins and stages of the SA and JA defence networks may indicate that pathogen-mediated selection pressure has driven pathway evolution.

Some pathogens deploy effectors that target and disable, deactivate, or modulate SA biosynthesis. The well-known chorismate mutase and isochorismatase effectors act to dampen SA signaling by diverting precursors away from the ICS and PAL salicylic biosynthetic routes, thoroughly reviewed by Bauters et al. (2021). *Xanthomonas campestris* effector XopD and *Verticillium dahliae* effector VdSCP41 reduce the expression of *ICS1* by inhibiting its transcriptional activators, therefore reducing the production of SA (Canonne et al., 2011). Other effectors, including F5 produced by the fungal pathogen *Mycosphaerella pinodes,* target the PAL biosynthetic pathway by inhibiting *PAL* and *CINNAMATE-4-HYROXYLASE (C4H)* biosynthetic genes (Hiramatsu et al., 1986). Considering this, it is plausible to hypothesize that the diversification and segregation of SA biosynthesis into two genetically and spatially unlinked routes (i.e., the ICS and PAL biosynthetic pathways) could be a measure employed to avoid pathogen-mediated defence disablement. Indeed the buffering of many defence hubs by way of redundancy has also been identified as a way to increase robustness of defence network against manipulation (Hillmer et al., 2017).

Individual proteins can also be the target of pathogen-mediated diversification. The JAZ repressor proteins multigene family consists of with 13 members displaying partial functional redundancy (Chini et al., 2007; Thines et al., 2007; Yan et al., 2007; Guo et al., 2018). As a key regulatory hub of the JA defence network, the JAZ proteins are the target of numerous effectors (thoroughly reviewed by Ceulemans et al., 2021). For example, the *Pseudomonas syringae* effector HopZ1a acetylates JAZ1 to promote its degradation, thereby activating the JA defence response (Jiang et al., 2013). In order to escape pathogen-mediated defence disablement, it is possible that the JAZ proteins underwent significant diversification and neofunctionalisation, thus further shaping pathway structure.

## Arabidopsis as a model species for horticulturally important crop species

Understanding how plants exist in multi-stressor environments is a developing field of research. Most studies have focused on a single species due to the complexities of the hormonal networks underpinning defence. Selected due to its compact size, annual lifecycle, and relatively small genome, the model plant *Arabidopsis thaliana* has been adopted as a convenient laboratory-based model to investigate the genetic determinants of defence (Koornneef and Meinke, 2010). Increasingly, recent studies have highlighted that the lessons learnt in model plants often require adjustment before being applicable to horticulturally important species, potentially due to different evolutionary drivers shaping their defence networks (De Vleesschauwer et al., 2014). The domestication of plants has driven the monoculturisation of crop species, selecting for favorable traits including shorter growing times, high yield, preferred taste profiles, and increased shelf life (Smýkal et al., 2018). It is likely that the selection for these agronomically important traits inadvertently selected for, or against, defence-associated traits, shaping the evolution of defence networks that are structurally different to species that have not been exposed to the same selection pressure(s), namely non-crop and model plant species (Fernandez et al., 2021). Furthermore, defence-associated genes appear to be particularly malleable to evolutionary drivers, demonstrated by the extensive functional divergence of defence genes in *Oryza* species (Zhang et al., 2014). Polyclonal wild populations of plants are able to retain a broad diversity of these factors whereas the monoclonal populations that predominate in agriculture carry a much more limited set, allowing pests and diseases to adapt to neutralize these targets more rapidly, often by targeting key hormone control points (Gimenez-Ibanez et al., 2014).

Plant lifecycle is potentially another important determinant of defence. Arabidopsis and other annual plants complete their lifecycle within a year whereas perennial plants, including most crop species, have extended lifespans spanning at least 2 years. As such, plants with shorter lifecycles may favor traits facilitating disease tolerance, allowing them to survive long enough to reproduce before succumbing to disease. In contrast, perennial plants may prioritize the development of disease resistance, facilitating the elimination of the stressor in order to survive multiple seasons (Newton, 2016; Pagán and García-Arenal, 2018).

Collectively, the domestication of crop species and the defence requirements of annual and perennial plants highlight the different evolutionary drivers shaping the defence networks of model and crop species. Several key differences in the structure and components of the SA and JA defence networks between Arabidopsis and other species have already been identified, notably in the SA biosynthetic pathways (Shine et al., 2016) and modification requirements of JA prior to pathway activation (Monte et al., 2018). To understand how plants in general perceive and respond to stress in the environment, the field must evolve to encompass non-model species across a diverse range of phylogenetic taxonomic divisions as well as abiotic stressors to gain a full picture of the complex scenarios plants navigate in the natural environment.

## Author contributions

ES searched the literature, drafted the manuscript, and constructed figures. ER, JJ, and MT provided suggestions for writing and edited the manuscript. All authors contributed to the article and approved the submitted version.

## Funding

Support for this publication was provided under the Growing Futures Programme “The lightest tread” funded by The New Zealand Institute for Plant and Food Research Limited from the bulk-funded Strategic Science Investment Fund from the New Zealand government to the institute.

## Conflict of interest

JJ, MT, and ER are employed by The New Zealand Institute for Plant and Food Research Limited.

The remaining authors declare that the research was conducted in the absence of any commercial or financial relationships that could be construed as a potential conflict of interest.

## Publisher’s note

All claims expressed in this article are solely those of the authors and do not necessarily represent those of their affiliated organizations, or those of the publisher, the editors and the reviewers. Any product that may be evaluated in this article, or claim that may be made by its manufacturer, is not guaranteed or endorsed by the publisher.
