# Supplementary material for: Comparison of the pathway structures influencing the temporal response of salicylate and jasmonate defence hormones in Arabidopsis thaliana
Source: Front Plant Sci. 2022 Sep 9;13:952301. doi: 10.3389/fpls.2022.952301 (PMC9504473; doi:10.3389/fpls.2022.952301)
Supplement: Supplementary file 1 [file Table_1.docx]

# Comparison of the pathway structures influencing the temporal response of salicylate and jasmonate defence hormones in *Arabidopsis thaliana*

# Erin.A. Stroud^1,2^, Jay Jayaraman^1,3^, Matthew.D. Templeton^1,2,3^, Erik.H.A. Rikkerink^1*^

^1^The New Zealand Institute for Plant and Food Research Limited, 120 Mount Albert Road, Auckland, New Zealand

^2^University of Auckland, Auckland, New Zealand

^3^Bioprotection Aotearoa, New Zealand

*** Correspondence:**Erik.H.A. Rikkerink
erik.rikkerink@plantandfood.co.nz

**Supplementary Table 1.** **Literature overview of the *Arabidopsis* transcriptomic response to different types of pest/pathogen attack.**

An overview of the findings of literature assaying the transcriptomic response of *Arabidopsis* to various pests and pathogens. The scope of this literature overview is limited to resistant *Arabidopsis*-microbe and *Arabidopsis*-insect interactions. The type of pest/pathogen (‘Pest/pathogen lifestyle’), details of the challenging pest/pathogen (‘Stimulus’), predicted major defence response pathway (‘Predicted hormone pathway’), type of disease assay and timing of disease development (‘Disease development assayed’), timing and characteristics of the defence response (‘Transcriptomic response’), and reference (‘Reference’) are provided for each study, where possible.

| Pest/pathogen lifestyle | Stimulus | Predicted phyto-hormone pathway | Disease development assayed | Transcriptomic response | Reference |
| --- | --- | --- | --- | --- | --- |
| Biotrophic pathogen | *Pseudomonas syringae* pv. tomato (DC3000 AvrRpt2) | Salicylic acid | Tissue collapse observed at 48 hpi | Transcriptomic response assayed with northern blot analysis and microarray. PR1 expression peaked at 12 hpi and gradually decreased at 48 and 72 hpi. Hormone accumulation initiated at 3 hpi before peaking at 24 hpi and returning to basal levels at 48 hpi. | de Vos *et al.*, 2005 |
| Biotrophic pathogen | *Pseudomonas syringae* pv. tomato (DC3000 AvrRpt2 and AvrRpm1) | Salicylic acid | Not assayed | Transcriptomic response assayed with RNA-sequencing. Response initiated at 3 hpi and returned to basal levels at 24 hpi. | Mine *et al.*, 2018 |

| Biotrophic pathogen | *Hyaloperonospora arabidopsidis* Emoy2 | Salicylic acid | Lesion development assayed at 2 – 5 dpi | Transcriptomic response of defence marker gene PR1 was assayed with qRT-PCR. Expression of PR1 peaked at 24 hpi before declining at 72 hpi and returning to near basal levels by 120 hpi. | Asai *et al.*, 2014 |
| --- | --- | --- | --- | --- | --- |
| Sucking insect (rasping) | *Frankliniella occidentalis* | Salicylic acid | Lesion development observed at 12 hpi | Transcriptomic response assayed with northern blot analysis and microarray. PDF1.2 expression was upregulated at 12 hpi and increased at 24 hpi. Hormone accumulation initiated at 12hpi and continued to increase at 72 hpi. | de Vos *et al.*, 2005 |

| Sucking insect (rasping) | *Frankliniella occidentalis* | Salicylic acid | Lesion development observed at 48 hpi | Transcriptomic response of defence marker genes were assayed with qRT-PCR. Jasmonic acid marker genes were upregulated at 5 hpi before peaking at 10 hpi and beginning to decline at 24 hpi. Salicylic acid marker genes were upregulated at 10 hpi – 24 hpi. | Abe *et al.*, 2008 |
| --- | --- | --- | --- | --- | --- |
| Sucking insect (stylet) | *Myzus persicae* | Salicylic acid | Lesion development assayed at 72 hpi but not observed | Transcriptomic response assayed with microarray. PR1, PDF1.2 and HEL expression were upregulated at 48 hpi and PR1 and HEL were upregulated at 72 hpi. | de Vos *et al.*, 2005 |

| Sucking insect (stylet) | *Myzus persicae* | Salicylic acid | Not assayed | Transcriptomic response assed with microarray. Salicylic acid pathway genes were upregulated at 6 hpi before peaking at 24 hpi and beginning to decline at 48 hpi. Jasmonic acid/ethylene pathway genes were strongly and stably induced at 6, 24, and 24 hpi. | Kerchev *et al.*, 2013 |
| --- | --- | --- | --- | --- | --- |
| Necrotrophic pathogen | *Alternaria brassicicola* | Jasmonic acid | Not assayed | Transcriptomic response of PDF1.2 was assayed with RNA gel blot analysis and found to be upregulated at 48 – 96 hpi. Protein accumulation of PDF initiated at ~ 50 hpi before peaking at ~75 hpi and remaining stable at 100 hpi. | Penninckx *et al.*, 1996 |

| Necrotrophic pathogen | *Alternaria brassicicola* | Jasmonic acid | Lesion development observed at 72 hpi | Transcriptomic response of defence marker genes was assayed with qRT-PCR. Transcriptomic response initiated at 12 hpi with jasmonic acid marker genes responsive between 24 – 36 hpi. | van Wees *et al.*, 2003 |
| --- | --- | --- | --- | --- | --- |
| Necrotrophic pathogen | *Alternaria brassicicola* | Jasmonic acid | Lesion development observed at 48 - 72 hpi | Transcriptomic response assayed with northern blot analysis and microarray. PDF1.2 upregulation initiated at 24 hpi and increased at 48 hpi. Hormone accumulation initiated at 12 hpi and gradually increased over time, peaking at 72 hpi. | de Vos *et al.*, 2005 |

| Necrotrophic pathogen | *Alternaria brassicicola* | Jasmonic acid | Lesion development observed at 48 hpi and assayed at 5 dpi | Transcriptomic response assayed with semi-quantitative RT PCR. The response of several jasmonic acid defence marker genes was assayed and found to be upregulated at 72 hpi. | Mukherjee *et al.*, 2009 |
| --- | --- | --- | --- | --- | --- |
| Necrotrophic pathogen | *Botrytis cinerea* | Jasmonic acid | Not assayed | Transcriptomic response assayed with microarray. Found that the transcriptomic response initiated at 16 hpi and increased at 48 hpi. | Ferrari *et al.*, 2007 |
| Necrotrophic pathogen | *Botrytis cinerea* | Jasmonic acid | Not assayed | Transcriptomic response assayed with RT-qPCR. PDF1.2 expression was initially upregulated at 14 hpi before increasing at 24 hpi and remaining stably upregulated at 48 hpi. | Birkenbihl *et al.*, 2012 |

| Necrotrophic pathogen | *Botrytis cinerea* | Jasmonic acid | Lesion development observed at 36 – 72 hpi | Transcriptomic response initiated at 14 hpi with jasmonic acid-mediated defence signalling becoming upregulated from 22 – 48 hpi. | Windram *et al.*, 2012 |
| --- | --- | --- | --- | --- | --- |
| Necrotrophic pathogen | *Botrytis cinerea* | Jasmonic acid | Not assayed | Transcriptomic response assayed with RNA-sequencing. Global transcriptomic response initiated at 18 hpi and increased at 24 hpi. | Coolen *et al.*, 2016 |
| Necrotrophic pathogen | *Botrytis cinerea* | Jasmonic acid | Not assayed | Transcriptomic response assayed with RT-qPCR. The expression of several defence marker genes was found to be upregulated at 16, 24, and 40 hpi | Veillet *et al.*, 2017 |

| Chewing insect | *Pieris rapae* | Jasmonic acid | Lesion development observed at 12 hpi | Transcriptomic response assayed with northern blot analysis. VSP2 expression was upregulated at 24 and 48 hpi. Hormone accumulation initiated at 3 hpi and gradually increased over time, peaking at 48 hpi. | de Vos *et al.*, 2005 |
| --- | --- | --- | --- | --- | --- |
| Chewing insect | *Pieris rapae* | Jasmonic acid | Not assayed | Transcriptomic response of defence marker gene VSP2 was assayed with qRT-PCR. VSP2 expression peaked at 24 hpi, began declining at 28 hpi, and returned to basal levels by 48 hpi. | Vos *et al.*, 2015 |
| Chewing insect | *Pieris rapae* | Jasmonic acid | Not assayed | Transcriptomic response assayed with RNA-sequencing. Global transcriptomic response initiated at 3 hpi and remained upregulated at 24 hpi. | Coolen *et al.*, 2016 |

**Supplementary Table References**

Abe, H., Ohnishi, J., Narusaka, M., Seo, S., Narusaka, Y., Tsuda, S., et al. (2008). Function of jasmonate in response and tolerance of Arabidopsis to thrip feeding. *Plant and Cell Physiology, 49*(1), 68-80. DOI:[10.1093/pcp/pcm168](http://dx.doi.org/10.1093/pcp/pcm168)

Asai, S., Rallapalli, G., Piquerez, S.J., Caillaud, M.C., Furzer, O.J., Ishaque, N., et al. (2014). Expression profiling during Arabidopsis/downy mildew interaction reveals a highly-expressed effector that attenuates responses to salicylic acid. *PLoS Pathogens 10*(10), e1004443. doi: 10.1371/journal.ppat.1004443

Birkenbihl, R. P., Celia Diezel, C., and Somssich, I.E. (2012). Arabidopsis WRKY33 is a key transcriptional regulator of hormonal and metabolic responses toward *Botrytis cinerea* infection. *Plant Physiology 59*(1), 266-285. doi: 10.1104/pp.111.192641.

Coolen, S., Proietti, S., Hickman, R., Davila Olivas, N. H., Huang, P., Van Verk, M. C., et al. (2016). Transcriptome dynamics of Arabidopsis during sequential biotic and abiotic stresses.*The Plant Journal, 86*(3), 249-267. doi:10.1111/tpj.13167

de Vos, M., van Oosten, V. R., van Poecke, R. M. P, van Pelt, J. A., Pozo Jimenez, M. J., Mueller, M. J., et al. (2005). Signal signature and transcriptome changes of Arabidopsis during pathogen and insect attack.*Molecular Plant-Microbe Interactions, 18*(9), 923. doi:10.1094/MPMI-18-0923

Ferrari, S., Galletti, R., Denoux, C., De Lorenzo, G., Ausubel, F. M., and Dewdney, J. (2007). Resistance to Botrytis cinerea induced in Arabidopsis by elicitors is independent of salicylic acid, ethylene, or jasmonate signaling but requires PHYTOALEXIN DEFICIENT3. Plant Physiology, *144* (1), 367–379. [doi.org/10.1104/pp.107.095596](https://doi.org/10.1104/pp.107.095596).

Kerchev, P., Karpinska, B., Morris, J., Hussain, A., Verrall, S., Hedley, P., et al. (2013). Vitamin C and the abscisic acid-insensitive 4 transcription factor are important determinants of aphid resistance in Arabidopsis. *Antioxidants & Redox Signaling. 18*(16), 2091–2105. doi:10.1089/ars.2012.5097.

Mine, A., Seyfferth, C., Kracher, B., Berens, M. L., Becker, D., and Tsuda, K. (2018). The defense phytohormone signaling network enables rapid, high-amplitude transcriptional reprogramming during effector-triggered immunity.*The Plant Cell, 30*(6), 1199-1219. doi:10.1105/tpc.17.00970

Mukherjee, A.K., Lev, S., Gepstein, S., and Horowitz, B.A. (2009). A compatible interaction of *Alternaria brassicicola* with *Arabidopsis thaliana* ecotype DiG: evidence for a specific transcriptional signature. *BMC Plant Biology* *9***,**(31), 1-11. [doi.org/10.1186/1471-2229-9-31](https://doi.org/10.1186/1471-2229-9-31)

Penninckx, I.A., Eggermont, K.,Terras, F.R., Thomma, B.P., De Samblanx, G.W., Buchala, A., et al. (1996). Pathogen-induced systemic activation of a plant defensin gene in Arabidopsis follows a salicylic acid-independent pathway. The Plant Cell 8(12), 2309-2323. [doi.org/10.1105/tpc.8.12.2309](https://doi.org/10.1105/tpc.8.12.2309).

van Wees., S.C.M., Chang, H-S., Zhu, T., and Glazebrook, J. (2003). Characterization of the early response of Arabidopsis to *Alternaria brassicicola* infection using expression profiling. *Plant Physiology 132*(2), 606–617. [doi.org/10.1104/pp.103.022186](https://doi.org/10.1104/pp.103.022186).

Veillet, F., Gaillard, C., Lemonnier, P., Coutos-Thévenot, P., and La Camera, S. (2017). The molecular dialogue between *Arabidopsis thaliana* and the necrotrophic fungus *Botrytis cinerea* leads to major changes in host carbon metabolism. *Scientific Reports* 7(1), 17121. doi: 10.1038/s41598-017-17413-y.

Vos, I. A., Moritz, L., Pieterse, C. M. J., and van Wees, S C M. (2015). Impact of hormonal crosstalk on plant resistance and fitness under multi-attacker conditions.*Frontiers in Plant Science, 6*, 639. doi:10.3389/fpls.2015.00639.

Windram, O., Madhou, P., McHattie, S., Hill, C., Hickman, R., Cooke, E., et al. (2012). Arabidopsis defense against *Botrytis cinerea*: chronology and regulation deciphered by high-resolution temporal transcriptomic analysis*. Plant Cell 24*(9), 3530-3557. doi: 10.1105/tpc.112.102046.
